# Sexual Plasticity and Self-Fertilization in the Sea Anemone *Aiptasia diaphana*

**DOI:** 10.1371/journal.pone.0011874

**Published:** 2010-07-29

**Authors:** Ami Schlesinger, Esti Kramarsky-Winter, Hanna Rosenfeld, Rachel Armoza-Zvoloni, Yossi Loya

**Affiliations:** 1 Department of Zoology, George S. Wise Faculty of Life Sciences, Tel Aviv University, Tel Aviv, Israel; 2 Israel Oceanographic and Limnological Research, National Center for Mariculture, Eilat, Israel; University of Alabama, United States of America

## Abstract

Traits that influence reproductive success and contribute to reproductive isolation in animal and plant populations are a central focus of evolutionary biology. In the present study we used an experimental approach to demonstrate the occurrence of environmental effects on sexual and asexual reproduction, and provide evidence for sexual plasticity and inter-clonal fertilization in laboratory-cultured lines of the sea anemone *Aiptasia diaphana*. We showed that in *A. diaphana*, both asexual reproduction by pedal laceration, and sexual reproduction have seasonal components. The rate of pedal laceration was ten-fold higher under summer photoperiod and water temperature conditions than under winter conditions. The onset of gametogenesis coincided with the rising water temperatures occurring in spring, and spawning occurred under parameters that emulated summer photoperiod and temperature conditions. In addition, we showed that under laboratory conditions, asexually produced clones derived from a single founder individual exhibit sexual plasticity, resulting in the development of both male and female individuals. Moreover, a single female founder produced not only males and females but also hermaphrodite individuals. We further demonstrated that *A. diaphana* can fertilize within and between clone lines, producing swimming planula larvae. These diverse reproductive strategies may explain the species success as invader of artificial marine substrates. We suggest that these diverse reproductive strategies, together with their unique evolutionary position, make *Aiptasia diaphana* an excellent model for studying the evolution of sex.

## Introduction

Traits that influence reproduction in animal and plant species have been a central focus of evolutionary biology since Darwin. Therefore, considering their evolutionarily basal position, the Anthozoa (Cnidaria) provide important models for further understanding processes affecting reproductive strategies in the eumetazoan (i.e., cnidarian-bilaterian) ancestor and in modern vertebrates [1]. Many anthozoans reproduce through both asexual and sexual means [2]. In general, populations of organisms that rely on asexual reproduction are ultimately characterized by the number of genetic individuals (genets) being lower than the number of actual individuals (ramets) in an area [3]. Indeed at times such local populations may be asexual products of single clones. If this is the case, that the possibility of fertilization occurring within a genet or clone (defined here as self-fertilizing or “selfing”) may be an important reproductive option for such organisms. The evolution of these species may be enhanced by the meiosis and recombination that occur during sexual reproduction [4]–[6] while asexual reproduction may enhance their successful occupation of new spaces [7]. In most anthozoans, successful sexual reproduction occurs when either both eggs and sperm are shed into the water column, where fertilization and development occur, or when only sperm are released and fertilization is internal [2]. Spawning events may occur as single yearly events or repeatedly throughout the year; as single species or mass multispecies events [8]–[9]. Thus, genetic variability may be attained via either reproductive isolation or possible hybridizations [10].

Sea anemones (Cnidaria: Anthozoa) exhibit a variety of patterns of sexual reproduction and breeding patterns, even among species of the same genus [11]. One such anemone is the sea anemone *Aiptasia diaphana* from the eastern Mediterranean Sea. This species is found primarily in isolated fouling communities, making it an excellent model for studying how reproductive strategies may be instrumental in establishing and maintaining new populations. We therefore, undertook to evaluate its reproductive modes in order to understand how they may reflect its dispersal strategy.

## Results

### Natural gametogenic period and sexual character

**Figure 1:**
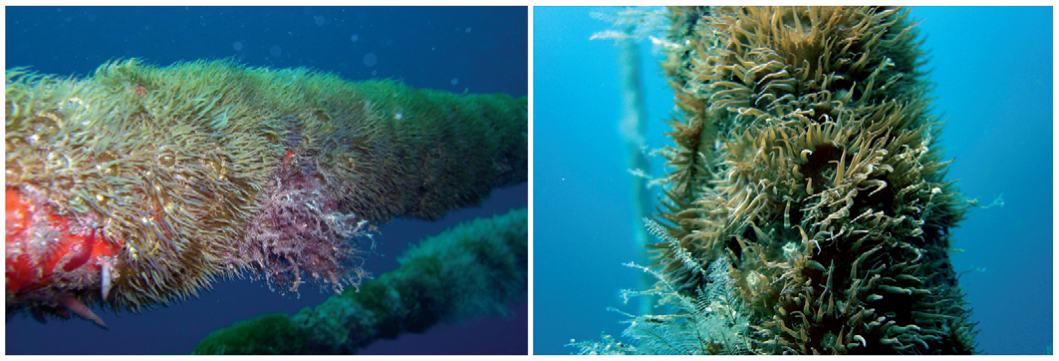
Field populations of *Aiptasia diaphana* forming a dense “carpet” on anchor rigs of an abandoned net pen fish farm 1.8 km offshore.

**Figure 2:**
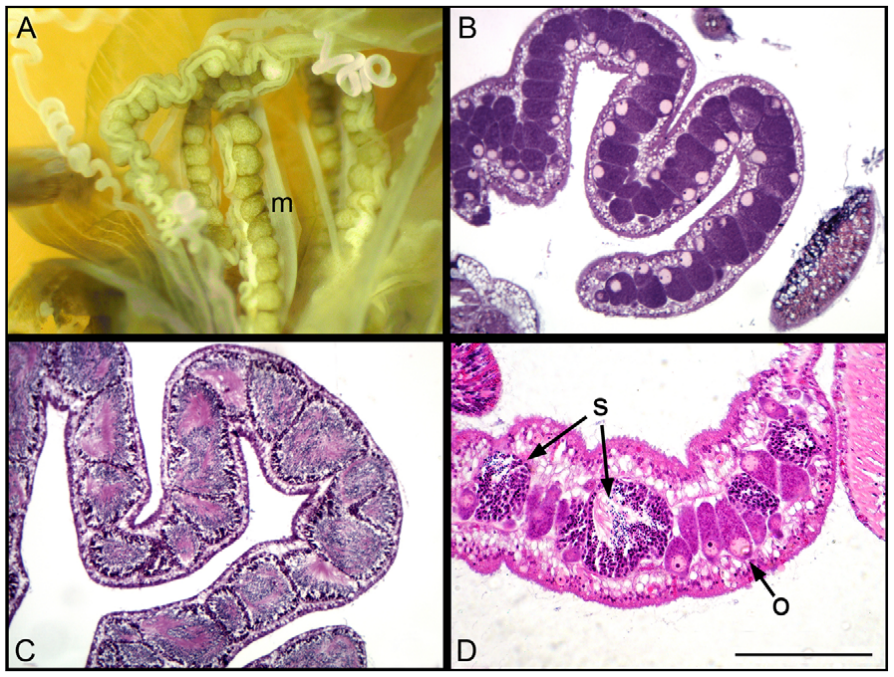
Anatomy and histology of gonads of *Aiptasia diaphana.* A) A dissected anemone illustrating the morphology of the gonads along the mesenteries (m) of the polyp. B) Histological section of a female gonad showing well developed oocytes with visible germinal vesicles and nucleoli. C) Histological section of a male gonad showing well developed spermaries. D) Histological section of a hermaphrodite gonad showing well developed oocytes (o) alongside interspersed spermaries (s) at various stages of development. Bar corresponds to 200 µm in B and to 100 µm C and in D.

In field populations of *Aiptasia diaphana* (Figure 1) gametogenesis occurred between April and August with a peak in the percent reproductive individuals (see Figure S1), as apparent from the presence of gametes in the histological sections analyzed (Figure 2). During the gametogenic season randomly sampled specimens were either male (n = 14) or female (n = 11), or did not possess gametes (n = 55). No hermaphrodites were found in samples of naturally occurring *A. diaphana*.

### Asexual reproduction rate

In laboratory culture, the rate of asexual reproduction was affected by controlled seasonal variations. Under June–August (summer) temperature–photoperiod conditions genets propagated *ca.* one order of magnitude more ramets (time = 77 d, *n* = 255±27) than under December – March (winter) conditions (t = 78 d, *n* = 23±12). The rate of asexual reproduction differed significantly between the summer and winter treatments (2 way ANOVA and Tukey's HSD test, *df* = 1 Fseason 83.61; p<0.05). Accordingly, each founder genet that produced 255 ramets (individuals resulting from asexual reproduction) during summer and in turn produces at least 20 additional ramets during winter can, theoretically, produce over 5,000 ramets (20_winter_X 255_summer_) over a one-year period.

### Sexual character of controlled laboratory-reared genets

Microscopic analysis of ramets (*n* = 304) sampled from six different laboratory-reared genet lines (G_1_ – G_6_), each founded by one individual of a known sex, revealed that the male phenotype was preserved throughout the experiment in one genet (G_5_) (Table 1, Figure 2). The other five genets gave rise to both female and male phenotypes, with a skewed female/male ratio in favor of the founder sex (Table 1). In addition, ramets derived from one female genet (G_2_), included not only males and females but also seven hermaphroditic individuals (Table 1).

**Table 1:** Sex of ramets derived from six different genets of known sex.

Genet	founder sex	n female ramets	n male ramets	n hermaphrodites	n total	% labile sex
G_1_	female	34	3	0	37	8%
G_2_		22	15	7	44	50%
G_3_		36	8	0	44	18%
Total		92	26	7	125	26%
G_4_	male	2	42	0	44	5%
G_5_		0	71	0	71	0%
G_6_		25	39	0	64	39%
Total		27	152	0	179	15%

The table depicts results of screening of a total of 304 ramets sampled from the six genets (G_1_-G_6_).

### Fertilization

**Figure 3:**
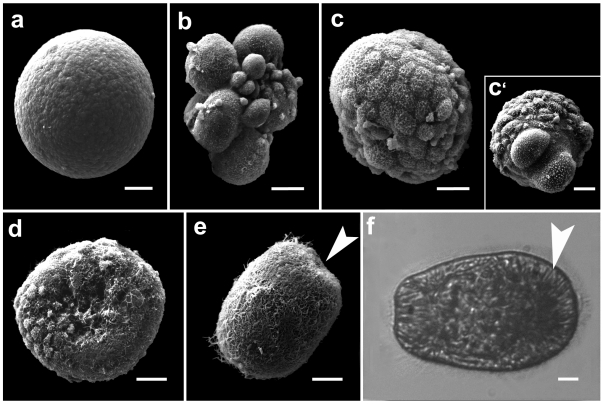
Development of self-fertilized gametes of the anemone *Aiptasia diaphana* (scale bars = 10 µm). (a) Released non fertilized oocyte (SEM). (b) late cleavage stage showing asynchronous “chaotic” cleavage (SEM). (c) Blastula from animal pole perspective, note small micromeres. (c') Blastula from the vegetal pole perspective, showing large yolky macromeres at the vegetal pole (SEM). (d) Prawn chip stage. (e) Gastrula. The blastopore is marked by an arrow. (SEM). (f) Swimming planula, nematocysts marked by arrowhead (light micrograph).

Gametes spawned by G_2_ (*n* = 3 spawn dates and G_3_ (*n* = 2 spawn dates) genet lines self-fertilized. Similarly, gametes spawned by G_1_cross-fertilized with gametes of G_5_ (*n* = 4 spawn dates). Zygotes derived from “selfing” and out-crossing developed into swimming planula larvae. The embryos underwent “chaotic” cleavage. On d-4 post-spawning, nematocysts had developed in all planulae (Figure 3).

## Discussion

Asexual reproduction in sessile Anthozoa is important as it provides a means of rapid colonization of a new habitat, as well as providing a means for supplying multiple copies of genotypes that have already proven successful under the prevailing conditions [2]. Although asexual reproduction results in a low genetic diversity in a clonally derived population, [3] it provides them the with the ability to quickly establish populations in newly available spaces. Indeed under the experimental conditions of this study, asexual reproduction allowed the quick establishment of founding sea anemone populations especially during summer temperature and photoperiod conditions The observed increased rate of pedal laceration in *A. diaphana* (tenfold higher) under summer temperature and photoperiod conditions, when compared to the rate under winter conditions, suggests that metabolic components affected by differences in irradiance and/or water temperature, may play a key role in regulating asexual propagation in this species. This is not surprising as previous studies have shown similar temperature and irradiance dependence of fission and pedal laceration rates in a number of anemone species [2], [12]–[13]. When sexual reproduction occurs in a population of organisms, meiosis and recombination events drive their genetic variability [4]–[6]. In most anthozoans, successful sexual reproduction occurs when either, both eggs and sperm are shed into the water column, where fertilization and development occur, or when only sperm are released and fertilization is internal [2], [14]. Spawning of gametes, whether occurring as single annual events, or repeatedly throughout the year as single species events or as mass multispecies events [8]–[9], results in increasing genetic variability [10]. In addition, since Anthozoa, like other cnidarians, lack sequestered germ-cell lines [15], genetic variations occur somatically, and do not affect the gametes, or may be directly incorporated into developing gametes. This may result in the production of genetic variants occurring within a line of asexually reproducing individuals. Thus, it is possible that sexual reproduction occurring within a clonal population (defined here as self-fertilizing or “selfing”) may be an important additional reproductive option for such organisms, particularly since this trait can provide “reproductive assurance” to these populations. Accordingly, the ability to “self” seems to be advantageous in species that colonize new areas and is associated with weedy and invasive species [16]. Thus, species found in isolated and ephemeral environments, where few founder genotypes are present, may utilize asexual reproduction as the primary mode of dispersion [17]–[18], and sexual reproduction occurring within the genet may provide enhanced fitness through recombination events [12]–[13], [17], [19]. Reproduction in the Cnidaria has both intrinsic and extrinsic components [9], [14], [20], and mechanisms controlling sex determination in these organisms are still poorly understood [14]. In some Cnidaria, environmental parameters such as water temperature or food availability play a role in this process [21]–[23]. For example, in *Hydra* sex determination has a temperature- dependent component and in the hydrozoan *Clytia* sp. it was found that temperature affects the sex of the hydromedusae [20]–[21]. Environmental stress such as pollution or physical damage have also been reported to cause reproductive disruption, resulting in changes in the sex of the organism [24], [25]. In addition to extrinsic conditions, intrinsic processes such as the age or size of an individual can also affect reproduction in these organisms. For example *Stylophora pistillata* colonies display solely male function at small sizes, but become simultaneous hermaphrodites once they exceed a species-specific threshold size [24]. When stressed, they revert to their male function [25]–[26]. Sexual plasticity is also found in some of the massive Faviid corals, with edge polyps in the colony being male or sterile and the more central polyps developing as hermaphrodites [27]. A similar positional segregation of sexes occurs in the clonally-produced aggregations of the tropical corallimorpharians of the genus *Rhodactis* [28]. Their sex changes from sterile or male to hermaphrodite or female as their position in the colony changes [22] Likewise, in the aggregating actinian *Anthopleura elegantissima*, mid-aggregate polyps are fertile and large, while edge polyps are mostly infertile and smaller [22]. Clear within-polyp sex lability was observed in polyps of solitary fungiid corals [29]. These corals display a bidirectional sex change, possibly as a response to energetic and/or environmental constraints and it was posited that, in these corals, sex change increases their overall fitness [29]. The ability of these Anthozoa to change sex as a result of environmental or physiological constraints reinforces the view that reproductive plasticity plays an important role in determining their ability to quickly occupy newly opened substrates. To date, the sea anemone genus *Aiptasia* is considered gonochoric, with male and female gonads residing in separate individuals and the sexes remaining distinct throughout life [11], [14], [23]. This finding was strengthened by our observations that no hermaphroditic specimens were identified in wild *A. diaphana* populations. However, the identification of several simultaneously hermaphrodite specimens (i.e., individuals containing both testicular and ovarian components in the same mesenteries; see Figure 2) among asexually derived descendent clones form a cultivated *A. diaphana* female genet line indicate that hermaphroditism although rare is possible in these organisms.

It is still unclear as to whether the hermaphroditism observed in the laboratory was induced by our manipulations or whether it is a common characteristic of this species which, due to its rarity, was overlooked by the implemented sampling program. The occurrence of hermaphroditism and of genet lines in which individuals may be of both sexes as well as of a stable male genet line among cultured *A. diaphana* populations, implicate possible tendencies toward androdiocey (i.e., populations consisting of males and hermaphrodites) though this remains to be verified. The observed sexual plasticity found in the cultured anemone populations, led us to question the advantages of such a phenotypic plasticity. We suggest that the intrinsic reproductive plasticity observed in clones of *A. diaphana* may enable an individual planula larva recruit to found and spatially dominate spaces newly available for settlement (Figure. 1) while still retaining the option for sexual reproduction even if all its neighbours are clones of the founder. The settled recruits proliferate asexually, producing numerous sexually- reproducing individuals that may in turn serve as new sources for larval dispersal. As a result of continual asexual propagation, genets of the sea anemone *A. diaphana* may be very long-lived, hypothetically resulting in the incorporation of large numbers of somatic mutations. Such mutations could become the driving force of genetic variability within a population.

On the other hand, relying solely on asexual reproduction may lead, to a severe reduction in genetic variation on short timescales and to limited dispersal of a species in space. One way to overcome this is for the founder organisms to reproduce clonally, and produce sexually plastic ramets that may be able to fertilize within the genet. Sexual reproduction between ramets found in isolated environments, like on the ropes in this study, though can only occur if there are no or few barriers to “selfing”. This supports Carlon's [30] suggestion that species with limited dispersal potential may have high rates of self-fertilization.

In marine species that reproduce by broadcast spawning (i.e., shedding of male and female gametes into the water column) and that rely on water currents and movement of sperm between individuals, the rapid dilution and relatively short lifespan of sperm means that outcrossed fertilization is proximity- dependent [31]. In sessile organisms in which the only proximate partner is a clone- mate, self-fertilization becomes the only method of achieving meiotically-driven genetic variability. Thus, genetic variability in the Anthozoa may be less dependent on sexual reproduction than on somatic mutations [2], [14], [32] that may be directly incorporated into the germ lines or gametes [1], [15], [33]. These genetic changes may then be passed on to the next generations. Mendelian inheritance patterns found in offspring resulting from sexual reproduction between clonemates (defined here as “selfing”) that may have undergone somatic mutations [34] may result in their benefiting from the evolutionary advantages of meiosis. Therefore, in clonal organisms self-fertilization remains an option when it is advantageous to do so. Thus the ability of individual clones to develop ramets of different sexes that can then outcross, fertilize within the clone or self-fertilize, may ultimately result in increase genetic variability via meiotic events even when fertilization occurs within the clone.

The occurrence of such sexual plasticity in clonal and colonial Anthozoa has led to questions regarding the possibility of fertilization within a clone i.e. “self-fertilization”. Indeed, “selfing” has been reported to occur in several hermaphroditic colonial coral species in both field experiments and molecular based studies [35]–[37]. In this study we experimentally manipulated the sea anemone clones, producing individuals of varying sex and inducing the occurrence of fertilization within the clone. This should not be surprising since, as first discussed by Darwin, [38], a major evolutionary advantage conferred by self-fertilization is the ability to reproduce when potential mates are limited, as occurred in our experimental setup. An important aspect of this reproductive capacity is the ability of these lineages to colonize new habitats from very small founding populations, as occurs in the laboratory reared *A. diaphana* populations. An example of the advantage of self-fertilization is that it occurs in founder organisms that colonize new areas, and is, therefore, associated with weedy and invasive species [16]. This advantage is especially clear in populations colonizing ephemeral substrates, where the chance of a founder individual encountering a conspecific is limited and short lived [34]. Self-fertilization, well known in plants, is one of the most important determinants of the genetic structure of flowering plant-populations [39]. Indeed, Kalisz et al [40]. showed that when the pollination environment in wild plant populations necessitates reproductive assurance, “selfing” rates increase. Those authors posited that reproductive assurance in unstable environments is achieved through mixed “mating” systems [40]. Thus, it is likely that, as seen in plants, the asexual reproduction together with the ability to self-fertilize found in the sea anemone *Aiptasia*, increases this organism's chances to successfully colonize new areas. Following the same rationale Ghiselin [41](suggested that selfing may occur when a population of organisms arrives at a critically low density where animals are sparse and cross-fertilization impossible. However, it is not yet clear whether specific endogenous mechanisms operate in these organisms that allow self-fertilization whenever optional outcrossing does not exist, or even when it does. Thus, the selfing observed in this study could have been triggered as a result of our experimental design, which emulated the establishment of *A. diaphana* populations from single founders. The evolutionary processes shaping the reproductive plasticity displayed in this organism are just beginning to be clarified. In view of the above, considering their evolutionarily-basal position in the Cnidaria [42] and their close resemblance to the eumetazoan (i.e., cnidarian-bilaterian) ancestor [1], sea anemones in general and *Aiptasia* in particular, may provide an excellent model for further clarification of the evolutionary consequences of sexual plasticity and self- fertilization.

## Materials and Methods

### Determination of sex and reproductive season in a natural population

Gonad development was monitored in natural populations of *Aiptasia diaphana*, situated 1.8 km off the Israeli Eastern Mediterranean coastline (32°24′9.85′′N, 34°50′49.92′′E), on anchor rigs of an abandoned net-pen fish farm (Figure 1). The site was reached from Michmoret Harbor (Israel) with an outboard motor boat. Sampling was carried out for a period of 16 months (from April 2003 to July 2004), at a depth of 7–25 m, at temperatures ranging from 15°C –30°C. Using SCUBA, between 5 and 10 specimens were carefully detached from the ropes with a scalpel. Specimens were collected from different ropes and different positions on the ropes. The collected specimens were then mixed in a collection container and five anemones were randomly sampled for histological analysis. They were relaxed by adding 10% MgCl_2_ SW to the container to a final concentration of 0.5% MgCl_2_ SW at ambient temperature, and then cooled to 4°C for 30 min, yielding unresponsive anemones, which would have fully recuperated if returned to SW. The relaxed anemones were fixed in 4% formaldehyde SW. After 24 h the fixative was removed and specimens were rinsed three times in water and placed in 70% ethanol. The fixed specimens were cut longitudinally and embedded in paraffin. The sections were used to prepare longitudinal and cross (one type from each half) histological sections (5 µm thick) of the anemone body column were prepared. Sections were stained using hematoxylin-eosin. Histological sections were analyzed under a light microscope to determine the presence and type of gonad. Fixed specimens were sent to Dr. O. Vicente (Universidad de Ceuta, Spain) for taxonomic classification, and deposited in The National Collections of Natural History, Tel-Aviv University. For each sampling period histological sections were prepared from the collected specimens and analyzed to determine the presence of gonads and type of gametes. The remaining specimens were maintained live, sexed and used as founders for further experimentation as described below.

### Controlled sea anemone culture system

**Figure 4:**
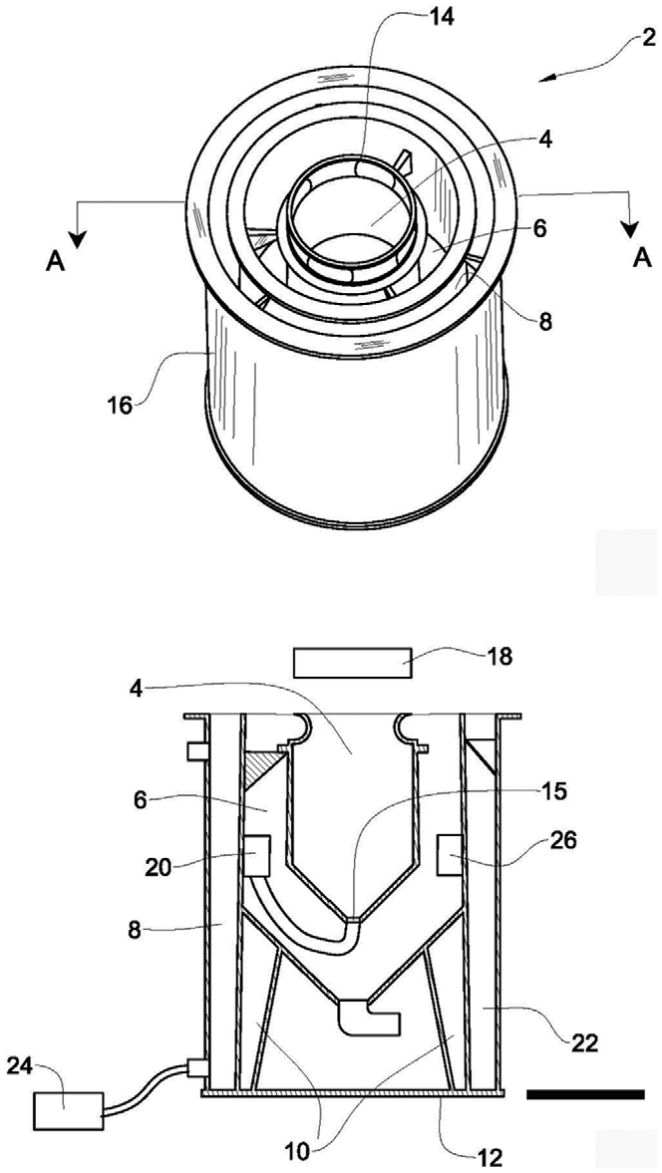
Modular culture apparatus [43]. The culture cell (4) is placed within a sedimentation chamber (6) which in turn is encompassed by a water jacket (8). A light source (18) is positioned above the culture cell. A pump (20) is fixed to a wall in the sedimentation chamber (6) and connected to the culture cell inlet port (15). Sediment is drained from the chamber via a waste outlet (22) connected to the bottom of the chamber. Culture temperature is controlled by the water jacket (8), using a water chiller (24) and a thermostat regulated heater (26). The legs (10) of the culture tank stand on the tank's bottom (12), and water outlets (14) are interspersed around the top of the cell in the water jacket tank (16). Scale bar = 20 cm.

To allow for controlled, uniform culture of *A. diaphana* (e.g., controlled temperature amplitude, Light:Dark cycle, water exchange, feeding, water flow), a culture apparatus (Figure 4) was set up indoors and connected to a flow-through seawater system. The sea water system provided a constant supply of filtered (5 µm) seawater (SW), which was evenly distributed between the culture tanks at a constant flow rate of 5 l h^−1^, via a pressure compensated dripper system (Ein-Tal^TM^, Israel). Genet lines were cultured on portable mesh fabric (SEFAR NITEX, Switzerland) anemone substratum in a 30 litre apparatus designed for the culture of benthic marine invertebrates [43]. Genet lines were cultured on portable mesh fabric (SEFAR NITEX, Switzerland) anemone substratum in a 30 litre apparatus designed for the culture of benthic marine invertebrates [43].

### *In situ* sexing of anemone founders and propagates

Sampled individual sea anemones were anesthetized in 0.5% MgCl_2_ -SW, and placed in glass Petri dishes under a Leica stereoscope and photodocumented using a Nikon DS5M camera connected to a PC enabling projection of the image on a 19 inch monitor. A small (5 mm) incision was made in the body wall using a surgical Wecker Spring Scissors (F.S.T, Germany). This allowed us to remove sections of the mesenteries by microdissection using the surgical spring scissors and thin Pasteur pipettes. For sex determination, a minimum of three 1 mM sections of mesenteries from each specimen were prepared as squashes on microscope slides analysed. Gamete type (oocyte/spermatid) was determined under a light microscope for sexually developed specimens. Male gonads were classified if sperm motility was observed, female gonads were classified if a germinal vesicle and nucleolus was clearly observed in the oocyte, hermaphrodites were classified if both oocytes and sperm were visible in the same individual. The sexed anemones were then placed in separate marked containers and allowed to recuperate prior to their use as founder individuals.

### Intra-genet sex ratio monitoring system

In order to initiate a genet line, one sea anemone of a determined sex (see above, termed founder) was settled on a new portable mesh (SEFAR NITEX, Switzerland) used as the anemone substratum, and placed in a clean new culture apparatus. Six genet lines were thus founded, three by males and three by females. An empty tank was connected to the drip system at the point closest to the entrance in order to control for recruitment and settlement of anemone from the sea via the filtered, open water circuit system. Photoperiod values were shifted weekly using data obtained from the Wise Observatory Astronomical Calendar, to coincide with ambient seasonal values. Light:Dark cycles were set using commercially available T5 fluorescent tubes (Aqua Medic ™ Reef White, Germany and ATI Blue Plus, Germany) set 20 cm above the anemone culture tanks, emitting an irradiance level of *ca.* 100 µE, temporally controlled by electronic controllers connected to the central electrical power circuits of the Marine Laboratory. After a twelve month period, ramets from each founder genet were sampled and their sex was determined as above.

### Quantification of asexual reproduction rate

To ascertain the rate of pedal laceration (asexual reproduction) of this anemone under laboratory conditions, we simultaneously tested the effect of summer (June – August) and winter (December – February) photoperiod and temperature amplitude as recorded in the Eastern Mediterranean (32°24′9N, 34°50′5E). Data were obtained from the Wise Observatory Astronomical Calendar and MEDATLAS/2002 database, MEDAR Group 2002). Culture temperature was controlled by a water jacket (Figure 4), using a 4 m^3^ h^−1^ water circulation pump, a thermostat regulated water chiller and thermostat regulated heaters [43]–[44].

Photo-period was controlled as described above, by weekly calibration of Light:Dark period, following Wise Observatory Astronomical Calendar data. Temperature amplitudes in the culture systems were calibrated weekly using the water-jacket temperature control system i.e., regulated heaters and chillers, to follow the seasonal temperature amplitude (MEDATLAS/2002, MEDAR Group 2002 database Figure S2). Six ramets from three genet lines (two ramets from each genet) were removed and settled on new substrata, each in a separate, sterilized culture tank. Thus, each genet was simultaneously exposed to two different temperature and photoperiod regimes (three culture tanks under summer conditions and three under winter conditions). Feeding regime, light quality and intensity, current character and water exchange were uniform in all culture tanks. The number of ramets in each culture tank was counted after 77–78 days following settlement of each founder. Data were analyzed using Student's t test for significance.

### Spawning and fertilization

Genets (*n* = 4) of *A. diaphana* were cultured in the indoor system described above (Figure 4), set up to emulate ambient seasonal conditions (i.e., light and temperature amplitude) of the natural reproductive season. Spawning was temporally controlled by shifting the photoperiod and water temperature (as described above) to match the ambient shifts that occur during the natural gametogenic period During peak reproduction the mesenteries, visible via the transparent body column, were milky white in appearance in males and an orange color in females.

To test for inter-genet and intra-genet fertilization, ramets of genet lines G_1_, G_2_, G_3_ and G_5_ were removed from the culture tanks prior to spawning and placed in sterile crystallizing dishes containing 0.22 µm filtered SW using the following setup. G_1_ (*n* = 4 100% male genet, Table 1) and female individuals (as identified by gonad color) from G_5_ (*n* = 4, mixed genet see Table 1) were placed in one crystallizing dish to test for inter-genet fertilization. To test for intra-genet fertilization, ramets from G_2_ (*n* = 7) and ramets from G_3_ (*n* = 7) were each placed in separate dishes. Each combination was repeated over three times. On the mornings following gamete release (spawning) gametes were siphoned into marked sterile glass Petri dishes containing 0.22 µm filtered SW. Developing zygotes were cultured in covered glass dishes at 26°C, 13:11 L:D cycle in an illuminated incubator (Binder, Germany). Development was monitored continuously. On d-4 post-fertilization, planula larvae were transferred to crystallizing dishes (SW) in the incubator.

## Supporting Information

Figure S1 Percent “wild” *Aiptasia diaphana* with gonads between April 2003 and July 2004.
(0.10 MB PDF)

Figure S2 Eastern Mediterranean (32°24′9N, 34°50′5E) temperature amplitude based data obtained from MEDATLAS/2002 database, MEDAR Group 2002.
(0.09 MB PDF)
