# Supplementary figures and images for: Sexual Plasticity and Self-Fertilization in the Sea Anemone Aiptasia diaphana
Source: PLoS One. 2010 Jul 29;5(7):e11874. doi: 10.1371/journal.pone.0011874 (PMC2912375; doi:10.1371/journal.pone.0011874)

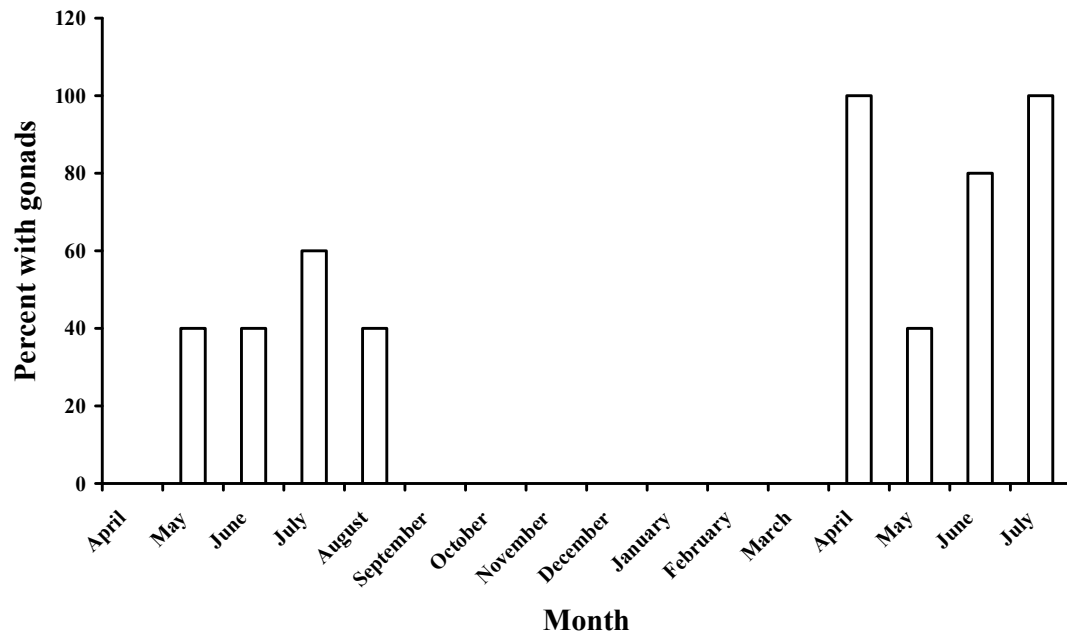

SFig 1 Percent of *Aiptasia diaphana* population with gonads sampled from the rope community

Supplement: Figure S1 — Percent “wild” Aiptasia diaphana with gonads between April 2003 and July 2004. (0.10 MB PDF) [file pone.0011874.s001.pdf]
