# Supplementary material for: Sexual Plasticity and Self-Fertilization in the Sea Anemone Aiptasia diaphana
Source: PLoS One. 2010 Jul 29;5(7):e11874. doi: 10.1371/journal.pone.0011874 (PMC2912375; doi:10.1371/journal.pone.0011874)
Supplement: Figure S2 — Eastern Mediterranean (32°24′9N, 34°50′5E) temperature amplitude based data obtained from MEDATLAS/2002 database, MEDAR Group 2002. (0.09 MB PDF) [file pone.0011874.s002.pdf]

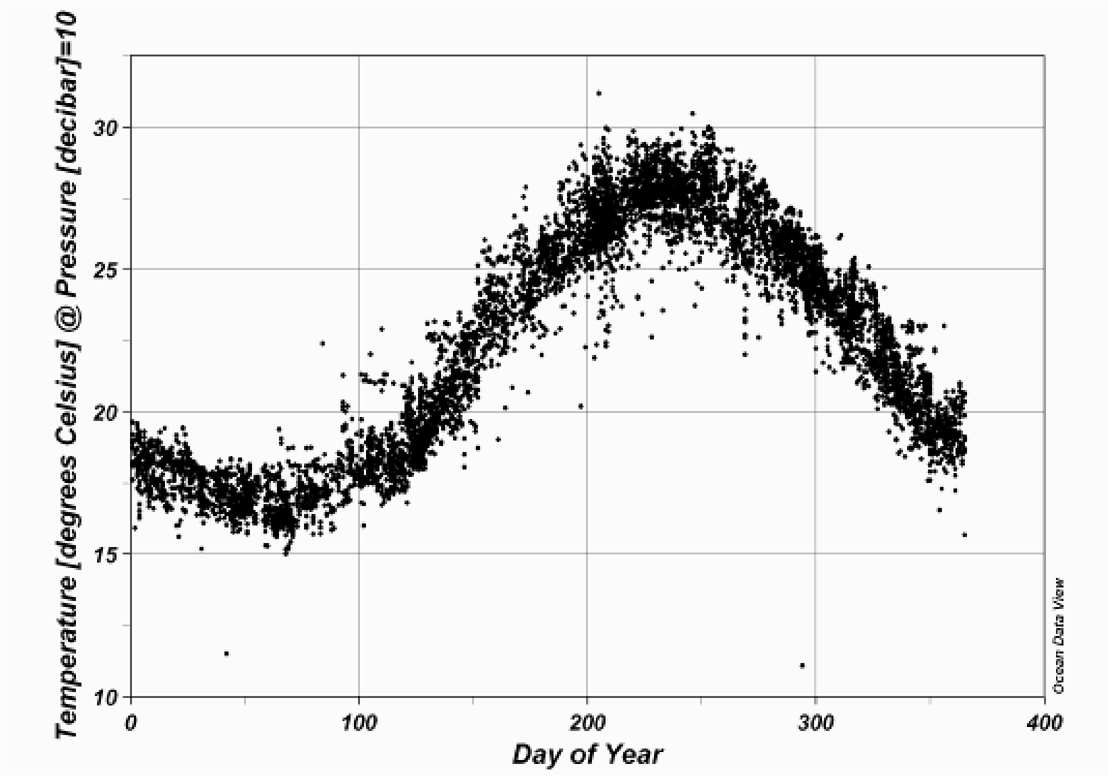

Figure S2 Eastern Mediterranean (32°24'9N, 34°50'5E) temperature amplitude based data obtained from MEDATLAS/2002 database, MEDAR Group 2002
